# *Octoblepharumperistomiruptum* (Octoblepharaceae) a new species from the Neotropics

**DOI:** 10.3897/phytokeys.164.51783

**Published:** 2020-10-21

**Authors:** Noris Salazar Allen, José A. Gudiño

**Affiliations:** 1 Smithsonian Tropical Research Institute Apartado 0843-03092, Balboa, Ancón Panama, Panama Smithsonian Tropical Research Institute Ancón Panama Panama

**Keywords:** Brazil, Bryophyta, fenestrate, Panama, peristome, reticulate

## Abstract

*Octoblepharumperistomiruptum*, a new species of moss in the family Octoblepharaceae from Panama and Brazil, is described and illustrated. The new species is characterised by plants with a reddish-purple colour particularly at the leaf bases, peristomes of eight teeth, each tooth composed of two rows of cells, fenestrate and usually completely separated at the base, strongly vertically striate-reticulate, some striations forked-like in shape. At the base of the teeth, some striations are horizontally orientated, poorly developed or absent, particularly on the cell wall that is rupturing in the separation of the vertical rows of the cells that form each tooth.

## Introduction

*Octoblepharum* Hedw. is a widely-distributed moss genus that is found in tropical and subtropical regions. The genus was erected by J. Hedwig in his *Species Muscorum Frondosorum* in 1801. It was described to include plants with single peristomes of eight teeth, capsules with an apophysis and autoicous gametangia (*flos masculus femineo*) (Hedwig 1801). The name derives from the Greek words *okto* (οξτο: eight) and *blepharis* (βλεπηαρισ: an eyelash), based on the eight peristome teeth of the type species, *O.albidum* Hedw., which was described from material collected by Swartz in Jamaica (Hedwig 1801). Of the 20 species recognised worldwide (see Salazar Allen and Chantanaorrapint 2018, for details on species distribution), eleven are reported for the Neotropics (Salazar Allen 1991, 1992, 1994). These are *O.albidum*, *O.ampullaceum* Mitt., *O.costatum* H.A. Crum, *O.cocuiense* Mitt., *O.cylindricum* Schimp. *ex* Mont., *O.erectifolium* Mitt., *O.leucobryoides* O. Yano, *O.pulvinatum* (Dozy & Molk.) Mitt., *O.rhaphidostegium* Müll. Hal., *O.stramineum* Mitt. and *O.tatei* (Williams) E.B. Bartram. The report of *O.africanum* from Brazil (Yano 1992) is doubtful as illustrations of the peristome do not correspond to the peristome described in the protologue (Cardot 1899), nor those observed by the senior author at (H). The status of *O.costatum* is also doubtful: based on the description by Crum (1983), Frahm (1994) suggested that this species could be a synonym of *O.cocuiense*.

Our taxonomic revision of numerous (over 300) neotropical specimens, thought to represent the pantropical *O.albidum*, showed very similar gametophytic morphology, but significantly distinct peristome structure and ornamentation. After reviewing specimens of *O.albidum* from the Province of Coclé in Panama, Brazil and other countries and relevant literature (Cardot 1899; Yano 1992; Salazar Allen 1992, 1994; Salazar Allen and Tan 2010; Salazar Allen and Chantanaorrapint 2018), we concluded that the examined material from the said specimens from Coclé in Panama and Brazil represent an undescribed species, which we formally describe below as *Octoblepharumperistomiruptum*.

## Materials and methods

From 2017–2019, we conducted morphological studies of fresh specimens from Panama and herbarium specimens from South America. Photographs of specimens in the field were taken with a LG K10, 2017 cell phone. Measurements of the morphological characters were made using a Leica-MZ6 stereomicroscope and an Olympus DPX50 light microscope. Microphotographs were obtained with an Olympus DP25 digital camera mounted on the latter microscope. Scanning electron micrographs (SEM) were made of samples from Panama and Brazil, following a modification of the methodology used by Salazar Allen (1993). Samples were viewed at different magnifications using a Zeiss Model Evo 40 vp SEM, with a backscattered electron detector and an acceleration voltage of 25 Kv, setting at the Smithsonian Tropical Research Institute (STRI), Panama. Digitised SEM images were post-processed and assembled in multipart figures using Adobe Photoshop.

## Taxonomic treatment

### Octoblepharum peristomiruptum

Taxon classificationPlantaeDicranalesDicranaceae

Salazar Allen & Gudiño sp. nov.

A9197D8C-ADB2-527B-8DF3-1F3C59B83D1B

Figs 1 , 2 , 3 , 4

#### Diagnosis.

*Octoblepharumperistomiruptum* is distinguished by its reddish to dark-purple coloured leaf bases, containing purple-coloured chlorocysts, with hyaline lamina 8–14 cells wide, unequally wide on each side of the costa, with purple cell walls, the exserted cylindrical capsule with long rostrate operculum and eight strongly vertically striate-reticulate teeth with the two rows of cells forming each tooth frequently separated at base.

**Figure 1.**
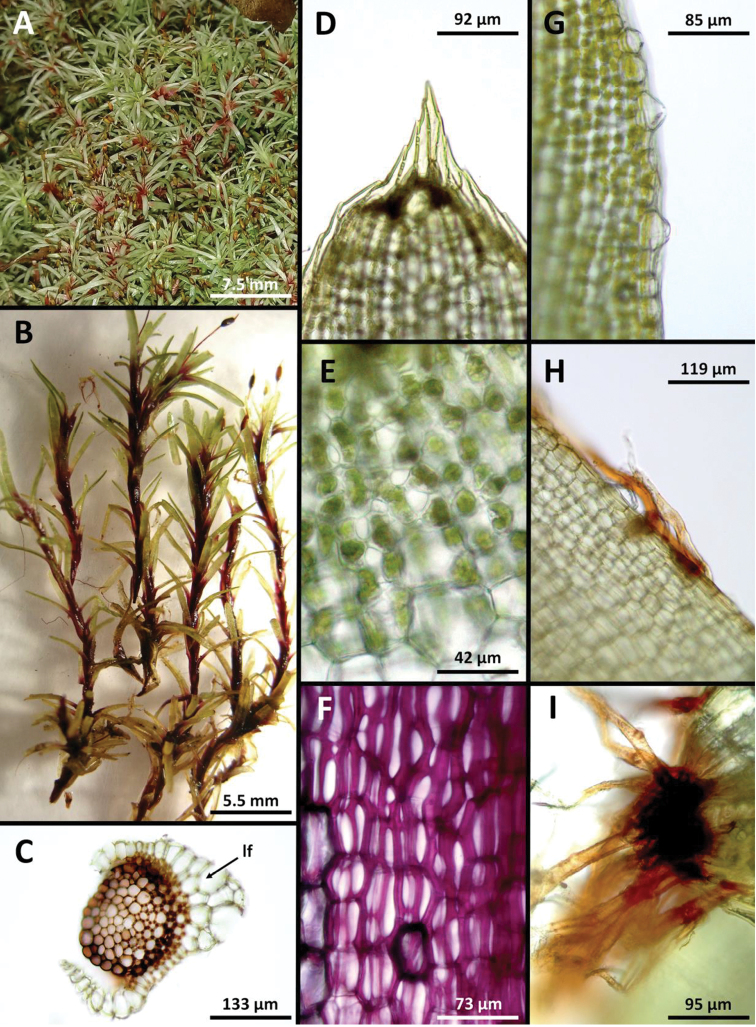
*Octoblepharumperistomiruptum* Salazar Allen & Gudiño. Photographs **A** habit in its natural environment **B** enlarged group of plants. Microphotographs **C** cross section of stem with young leaf (**lf**) **D** apex of leaf **E** chlorocysts near apex of leaf **F** chlorocysts at base of leaf (note the strong reddish-purple colouration) **G** enlarged hyalocysts on leaf border **H** rhizoids originating from border of leaf chlorocysts **I** rhizoids at apex of leaf. All from *Gudiño 3519* (PMA).

#### Type.

Panama. Coclé: Distrito de Penonomé, above Chiguirí Arriba, Mariposario Cerro La Vieja, 8°39.88'N, 80°12.07'W, 360 m alt., 1 Jan 2019, *J.A. Gudiño L. 3519* (holotype: PMA!; isotypes H!, NY!).

**Figure 2.**
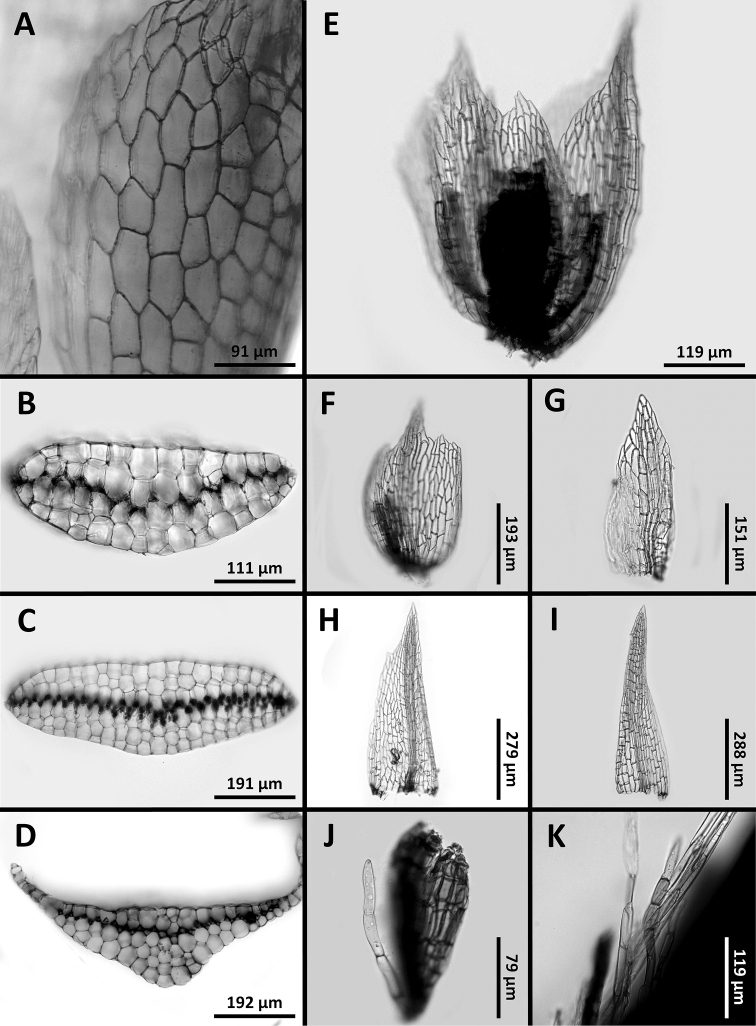
*Octoblepharumperistomiruptum* Salazar Allen & Gudiño. Microphotographs **A** leaf, hyaline lamina**B–D** cross sections of leaf **B** near apex **C** at mid-leaf **D** at base **E** androecium **F–I** male bracts **J** antheridium and paraphysis **K** paraphyses of gynoecium **A** taken from *Kulhmann 1621* (NY), **B–K** taken from *Gudiño 3519* (PMA).

#### Description.

***Plants*** (2.3–)3.5–5.0 cm tall. ***Stems*** erect, lacking a central strand of differentiated cells and thick-walled border cells, slightly tomentose at base, branching monopodial (pseudodichotomous), innovations arising early, during development of sporophyte. ***Rhizoids*** dark orange-red, arising from stem and leaves. Leaves ligulate, dentate in distal half, smooth at base, erect to slightly reflexed in upper third, (4.0–)5.5–6.7(–8.0) mm long, (0.4–)0.5–1.2 mm wide at base, including hyaline lamina; apex apiculate, ending in an elongate cell flanked by 2 hexagonal cells; margins of leaf slightly undulate due to swollen hyalocysts, these single or in groups of 2–3 cells. ***Limbidium*** extending from leaf apex to mid-leaf, 2(–3) cells wide, thinner at base. ***Hyalinelamina*** adpressed to stem, unistratose, composed of thin-walled pitted hyalocysts, the hyalocysts long and hexagonal at apex, hexagonal to pentagonal at mid-lamina next to costa and rectangular, quadrate and short pentagonal basally. ***Costa*** in cross-section composed of ventral and dorsal porose hyalocysts supporting a unistratose, median network of small, thick-walled chlorocysts, in cross-section chlorocysts forming an irregularly zig-zag row, quadrate to triangular at base and triangular to tear-shaped above; the chlorocysts at leaf base, below hyaline lamina, surrounded by one layer of porose hyalocysts ventrally and three layers dorsally, at hyaline lamina hyalocysts in 1–2 rows ventrally and 4 rows dorsally, at mid-leaf in 3 layers ventrally and 3–4 layers dorsally, near apex 2 layers ventrally and two dorsally. ***Autoicous***, perigonia axillary in short branches below the archegonia, antheridia surrounded by 5–7 small, mostly hyaline leaves (in some, only the central area of the leaf with chlorocysts), paraphyses 4–5 cells long with 1–2 brown basal cells, perichaetia terminal, archegonia with paraphyses to 10 cells long and with 1–2 short brown basal cells. ***Setae*** dark orange-red, smooth, sinistrorse, 4–5 mm long. ***Capsules*** dark red when mature, cylindrical (1.3–)1.6–2.0 mm long, the exothecial cells at mouth of capsule quadrate, dark red with slightly thickened transversal walls, at mid-capsule rectangular and quadrate with dark orange, thick longitudinal walls and thin transversal walls, (56–)80–92(–115) µm, becoming shorter towards mouth of capsule (26–)32–44 µm, phaneroporous stomata present at base of capsule. ***Prostome*** present. ***Peristome*** of eight elongate triangular teeth, inserted in mouth of capsule, each tooth composed of 2 rows of cells, basally fenestrate, strongly striate vertically, sometimes striations horizontally orientated at base and fading or absent in areas where separation of the rows of cells composing the tooth occurs. ***Operculum*** conic, long – rostrate, slightly curved. ***Calyptra*** cucullate, apex dark red, beige below. ***Spores*** brown, spheroid, densely gemmate, 14–16 µm.

**Figure 3.**
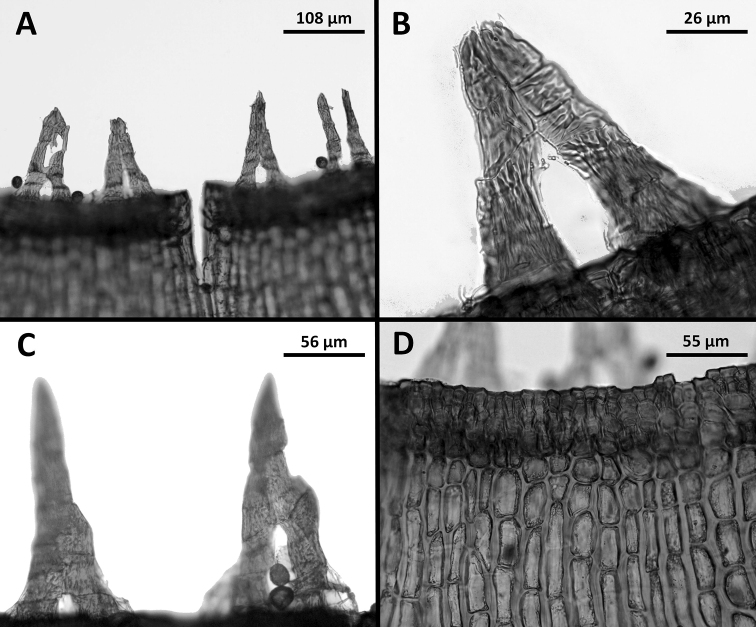
*Octoblepharumperistomiruptum* Salazar Allen & Gudiño. Microphotographs **A** four peristome teeth **B** dorsal view of peristome teeth with the two vertical rows of cells composing the teeth separating at base **C** two teeth with early and late separation of the two rows of cells **D** border and upper exothecial cells of capsule. **A, B, D** taken from *Occhioni 668* (H), **C** taken from *Gudiño 3519* (PMA).

#### Additional specimens examined (paratypes).

Brazil. Pará: Belem, Museu Goeldi, 29 Aug 1927, *P. Occhioni 668* (H), Belem, 13 Aug 1923, *J.G. Kulmann s.n.* (HBR-H), 1½ hr. upstream from Lageira airstrip, on Rio Maicuru, 0°55'S, 54°26'W, 243.84 m alt., 23 Jul 1981, *J.J. Strudwick & G.L. Sobel 3443* (NY); Matto Grosso: Pacca Nova, affl. do Mamoré, 23 Sept 1923, *J.G. Kulmann 516* (HBR-H).

#### Habitat, distribution and phenology.

*Octoblepharumperistomiruptum* was found on the cortex of a shrub, at 2 m above soil level in a private butterfly garden in the Coclé Province, Panama. The site is on the edge of the road. The climate in this area is characterised by average temperatures ranging from 23–30 °C (http://www.accuweather.com, accessed Jan 2020). In Brazil, the plant was found in three sites, on a living tree trunk in the “Museu Goeldi” reserve in Belem (Pará), on the trunk of a palm tree in a forested area in Matto Grosso and on a living tree trunk in a seasonally-flooded (varzea) forest. It is distributed in southern and northern Brazil and Central America (Panama). Plants with sporophytes were collected in Panama in January and, in Brazil in July, August and September.

#### Eponymy.

The species name refers to the character of the peristome teeth that rupture at base separating the two rows of cells that compose each tooth.

#### Conservation status.

The new species has been found in Coclé Province, Panama in a private conservation site and in the State of Pará, Brazil, on trees in the garden of the “Museu Goeldi” that is considered a reserve site. The conservation status of the other collection sites in Brazil is unknown. It is most probable that the species also occurs in other Central and South American countries. Given the limited knowledge of the current state of the sites where collections were made, the conservation status cannot be properly assessed. Thus, this new species is temporarily considered Data Deficient (DD).

**Figure 4.**
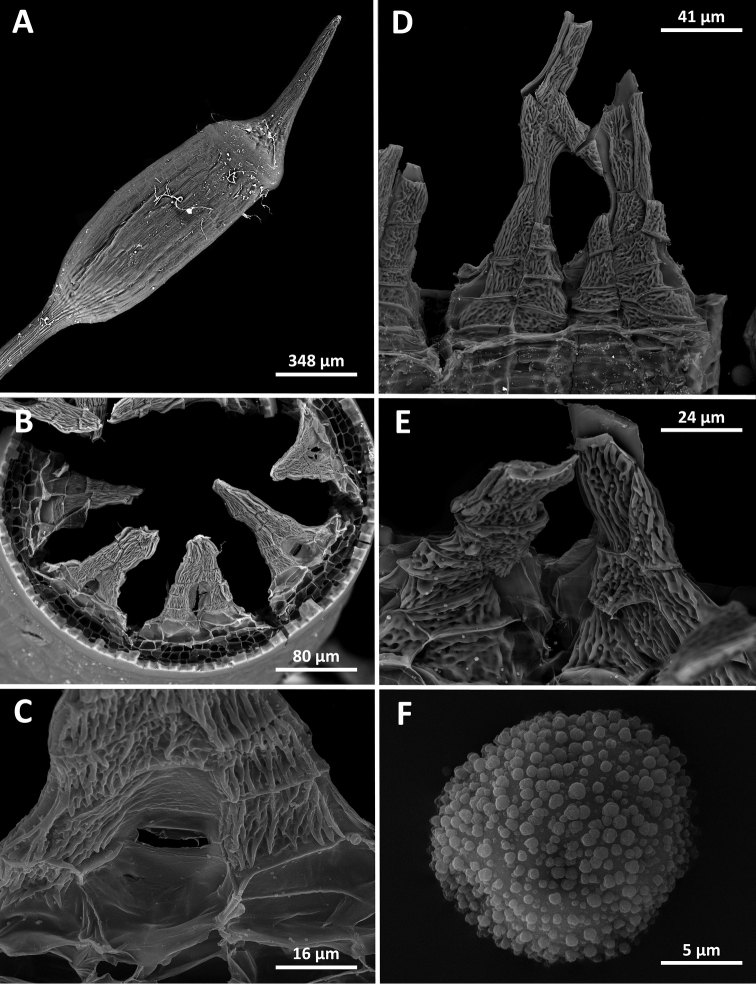
*Octoblepharumperistomiruptum* Salazar Allen & Gudiño. Scanning electron microscopy micrographs **A** sporophyte **B** peristome teeth dorsal view **C** close-up of rupturing wall **D** ventral view of two teeth **E** ventral view at base of two teeth **F** spore **A–C, E, F** taken from *Gudiño 3519* (PMA), **D** taken from *Strudwick & Sobel 3443* (NY).

## Discussion

*Octoblepharumperistomiruptum* is characterised by its tall habit, reddish stems, leaves with a strong dark reddish-purple colour at the base, exserted setae, cylindrical capsules and eight vertically striate-reticulate peristome teeth, each with two rows of cells separating at the base. Amongst other *Octoblepharum* species with eight peristome teeth, some populations of *O.albidum* have slightly pink-coloured leaves, 4–6(–8) mm long, but with the peristome mostly smooth or faintly striate, unlike *O.peristomiruptum*. Furthermore, Salazar Allen (1992) reported another *O.albidum* specimen (*Salazar* # *6588*) with red-purple leaves and strongly striate teeth with pronounced trabeculae, but this latter character is not present in *O.peristomiruptum*. Peristome teeth in most populations of *O.albidum* studied are solid, although sometimes they may have perforations at the base, as reported by Yano (1992). These perforations, however, are not as pronounced as those of *O.peristomiruptum*. Finally, the recent segregation of a new *Octoblepharum* species, previously included in *O.albidum* by Salazar Allen and Chantanaorrapint (2018), indicates that the specimen recorded in Salazar Allen (1992) needs further examination to ascertain its proper taxonomic status. *Octoblepharumalbidum*, as currently construed, might comprise a complex of cryptic species sharing similar gametophytic morphology, but with distinctive peristome structures and ornamentation (Salazar Allen and Chantanaorrapint 2018).

Other species of *Octoblepharum* with eight peristome teeth are *O.ampullaceum*, *O.benitotanii* Salazar Allen & Chantanaorr., *O.cylindricum*, *O.erectifolium*, *O pocsii* Magill & B.H. Allen and *O.rhaphidostegium*. Compared to *O.peristomiruptum*, the leaves of *O.ampullaceum* are longer (7–10 mm), tumid and the peristome is composed of eight pairs of slender, smooth teeth (Yano 1992). *Octoblepharumbenitotanii*, an Asiatic species, has shorter leaves (4.5–5.5 mm) with a prominent apiculus and the peristome has teeth with strongly foveolate-reticulate ornamentation on both surfaces and faint trabeculae (Salazar Allen and Chantanaorrapint 2018). *Octoblepharumcylindricum* has longer leaves (6–12 mm) with a light pink colouration at the base, the sporophyte has a long seta (10–18 mm) and the peristome teeth have a thickened mid-line and prominent trabeculae (Salazar Allen 1994). *Octoblepharumerectifolium* gametophytes have longer (15–25 mm), fragile leaves (Yano 1992) and the sporophyte has a long seta (to 16 mm) and eight elongate peristome teeth with pronounced trabeculae and reticulate ornamentation (Salazar Allen 1994). *Octoblepharumpocsii* is an African species with longer leaves (10–13 mm) and a peristome of short, fragile and smooth teeth (Magill and Allen 2013). *Octoblepharumrhaphidostegium* is dioicous, rather than monoicous as in *O.peristomiruptum* and the other species discussed above and it has a peristome with faint vertical striations, prominent trabeculae and a thickened mid-line (Müller 1895).

## Supplementary Material

XML Treatment for Octoblepharum peristomiruptum

## References

[B1] Cardot J (1899) Nouvelle classification des Leucobryacées. Revue Bryologique 26: 1–6. https://data.bnf.fr/en/12342667/jules_cardot/

[B2] Crum HA (1983) *Octoblepharumcostatum*. In: Magdefrau K (Ed.) The bryophyte vegetation of the forests and paramos of Venezuela and Colombia.Nova Hedwigia 38: 1–54.

[B3] Frahm J-P (1994) A contribution to the bryoflora of the Chocó region, Colombia. I. Mosses. Tropical Bryology 9: 89–110. 10.11646/bde.9.1.13

[B4] Hedwig J (1801) Species muscorum frondosorum descriptae et tabulis aeneis Ixxvii coloratis illustratae. Opus posthumum editum a Frederico Schwaegrichen Lipsiae [Leipzig], 50 pp. 10.5962/bhl.title.26

[B5] Magill R Allen BH (2013) *Octoblepharumpocsii*, sp. nov. a fragile- and long-leaved species in the *O.albidum* – complex from Africa. Polish Botanical Journal 58(1): 45–47. 10.2478/pbj-2013-0004

[B6] Müller K (1895) *Octoblepharumrhaphidostegium* n.sp. In: Brotherus VF (Ed.) Beiträge zur Kenntniss der brasilianischen Mossflora.Hedwigia 34: 1–119.

[B7] Salazar Allen N (1991) A preliminary treatment of the Central American species of *Octoblepharum* (Musci, Calymperaceae). Tropical Bryology 4: 85–97. 10.11646/bde.4.1.10

[B8] Salazar Allen N (1992) Notas para la revisión de las especies de *Octoblepharum* del neotrópico. Tropical Bryology 6: 171–179. 10.11646/bde.6.1.20

[B9] Salazar Allen N (1993) A Revision of the Pantropical Moss Genus *Leucophanes*. Brid. J. Cramer, Berlin, Germany, 281 pp.

[B10] Salazar Allen N (1994) *Octoblepharum*. In: Allen BH (Ed.) Moss flora of Central America. Part 1. Sphagnaceae–Calymperaceae.Monographs in Systematic Botany, Missouri Botanical Garden 49: 182–189.

[B11] Salazar Allen N Chantanaorrapint S (2018) *Octoblepharumbenitotanii* (Octoblepharaceae) a new species from the Old World tropics. Philippine Journal of Systematic Biology 12: 58–66. 10.26757/pjsb.2018a12005

[B12] Salazar Allen N Tan B (2010) *Octoblepharumarthrocormoides* (Calymperaceae) N. Salazar Allen & B.C. Tan, sp. nov., a new species from tropical Asia. Botany 88: 439–442. 10.1139/B10-022

[B13] Yano O (1992) *Octoblepharum* Hedw. Leucobryaceae (Bryopsida) do Brasil. PhD Thesis, Universidade de São Paulo, Brazil, 158–160, 209–213.

